# Molecular detection of SARS-CoV-2 infection in three geo-political zones of Nigeria: a cross-sectional study

**DOI:** 10.11604/pamj.2023.44.146.37400

**Published:** 2023-03-27

**Authors:** David Olufemi Olaleye, Adewale Victor Opayele, Hyacinth Chukwuebuka Egbuna, Adedayo Omotayo Faneye, Babatunde Adebiyi Olusola, Timothy Segun, Elizabeth Chibuzo Odeh, Magbagbeola David Dairo, Adeniyi Francis Fagbamigbe, Musa Adamu Sarki, Maryam Aminu, Ademola Johnson Ajuwon, Olasumbo Ganiyu Arinola, Chris Ezeora Achi, Sunday Aremu Omilabu, Georgina Njideka Odaibo

**Affiliations:** 1 Department of Virology, College of Medicine, University of Ibadan, Ibadan, Nigeria,; 2 Department of Public Health, Ministry of Health, Imo State, Nigeria,; 3 Department of Medical Microbiology and Parasitology, College of Medicine, University of Lagos, Lagos, Nigeria,; 4 Federal University Teaching Hospital, Abakaliki, Ebonyi State, Nigeria,; 5 Department of Epidemiology and Medical Statistics, College of Medicine, University of Ibadan, Ibadan, Nigeria,; 6 Amino Kano University Teaching Hospital, Kano, Nigeria,; 7 Department of Microbiology, Ahmadu Bello University, Zaria, Nigeria,; 8 Department of Health Promotion and Education, College of Medicine, University of Ibadan, Ibadan, Nigeria,; 9 Department of Immunology, College of Medicine, University of Ibadan, Ibadan, Nigeria,; 10 Ebonyi State Ministry of Health, Eboni, Nigeria

**Keywords:** COVID-19, SARS-CoV-2 infection, markets, Nigeria

## Abstract

**Introduction:**

sequel to the emergence of the Severe Acute Respiratory Syndrome Coronavirus 2 (SARS-CoV-2) and its subsequent spread to all continents of the world, humans have continued to experience severe devastation to their health and economies. To control the spread of this virus, it is important to detect the infection in recently infected and asymptomatic individuals who are capable of infecting others. This study was designed to detect ongoing SARS-CoV-2 Infection among asymptomatic individuals in open markets across three geopolitical zones in Nigeria.

**Methods:**

nasal and oropharyngeal swab samples were collected from 2,158 study participants between December 20^th^, 2020 and March 20^th^, 2021 from large open markets across three geo-political zones (Southwest, Northwest and Southeast) of Nigeria. Virus RNA was extracted from these swab samples and real time reverse transcription polymerase chain reaction (RT-PCR) was carried out for the detection of SARS-CoV-2 specific genes. Data were analysed using descriptive statistics.

**Results:**

a total of 163 (7.6%) of the 2,158 participants enrolled for the study tested positive for SARS-CoV-2 by RT-PCR. The rate of infection was significantly higher in the North-western States of the country when compared to the western and Eastern regions (P=0.000). Similarly, the rate of infection was higher among buyers than sellers (P=0.000) and among males when compared with females, though the difference was not significant (p=0.31).

**Conclusion:**

this study shows that there is a continuous spread of SARS-CoV-2, especially among active, asymptomatic individuals across many States in the country. There is therefore need to continuously educate citizens on the need to adhere to both the non-pharmaceutical and pharmaceutical preventive measures to protect themselves and ultimately curb the spread of the virus.

## Introduction

An outbreak of a severe respiratory disease started in Wuhan, China in December 2019 [1]. This epidemic later spread to become one of the most devastating global pandemics in recent times, leading to 258,164,425 confirmed cases and 5,166,192 deaths as at 25^th^ November 2021 [2]. The Severe Acute Respiratory Syndrome Coronavirus 2 (SARS-CoV-2) as it is now known is caused by a Coronavirus in the *Coronaviridae* family with an RNA genome [3]. In the recent past, notable coronaviruses that have caused significant morbidity and mortality in human population include the severe acute respiratory syndrome coronavirus (SARS-CoV) and the Middle East respiratory syndrome coronavirus (MERS-CoV), both of which are capable of causing acute lung injury and acute respiratory distress syndrome [4]. The ongoing SARS-CoV-2 pandemic has also been characterised by a series of clinical presentations ranging from asymptomatic to fatal pneumonia [5,6].

After its recent emergence in human population, the SARS-CoV-2 has spread rapidly across all continents of the world, infecting millions of people across America, Europe, Asia, the Pacific, Mediterranean and Africa [2]. To curb this spread, several control measures including travel restrictions, lockdowns, closure of public facilities, strict hand hygiene practices, wearing of facemasks, physical distancing and vaccination have been enacted [7]. These control measures have recorded varying degree of success due to limited compliance and the emergence of new fast-spreading SARS-CoV-2 mutant strains [8,9]. Surprisingly, in Africa, the epidemiological picture of the virus infection is different from other regions of the world in terms of the lower numbers of morbidity and mortalities from the disease. Despite this, the continent has reported over 152,000 deaths, majorly from South Africa while other African countries are still struggling to control the pandemic [2]. In Nigeria, the first case of SARS-CoV-2 was reported in February, 2020 and since then, over 254,000 cases have been reported with about 3000 deaths. Currently, Nigeria ranks fourth among countries with the highest number of confirmed cases of the virus infection on the continent [10].

Antibody-based detection tests may be utilized for surveillance purpose, whereas, viral antigen detection and viral nucleic acid-based real-time PCR assays are advised for diagnosis of suspected SARS-CoV-2 cases. Following appropriate laboratory detection, the necessary treatment and management follow-up procedures are carried out to avert further community spread. Furthermore, the viral antigen or nucleic acid-based technique affords early virus detection which is very valuable in the control of the pandemic [11].

Considering the impact that SARS-CoV-2 virus may have in a country as populous as Nigeria, in terms of morbidity and mortality, the importance of early detection of new and active cases in the control of the pandemic cannot be overemphasised. This is even more important among asymptomatic individuals who may be incubating and shedding the virus in their oral and respiratory droplets, thereby capable of infecting others. In view of the aforementioned, it is necessary to carry out surveillance activities to detect the presence of the virus, especially among clusters of people in the community [12]. This study was therefore carried out to detect ongoing SARS-CoV-2 virus Infection at the community level in three Geo-Political zones of Nigeria.

## Methods

**Figure 1:**
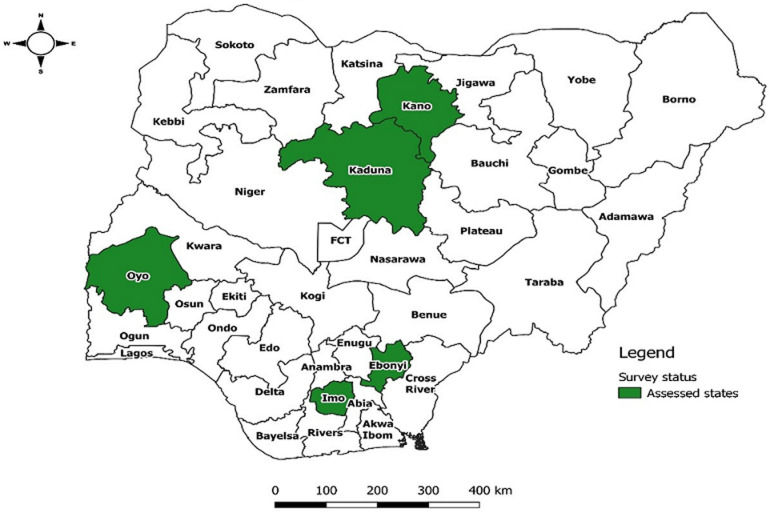
map of Nigeria showing states in the 3 geopolitical zones where study was conducted

**Study design and settings:** this cross-sectional study was carried out in open markets across three geo-political zones of Nigeria namely; the North-West (NW), South-West (SW) and South-East (SE). Figure 1 shows the States where participants were enrolled for the study. The selection of study areas was purposive based on the large human population in each of these cities in the respective States and geographical regions: Northwest Region (Kano in Kano State- 3,626,068, Kaduna in Kaduna State - 1,582,102), Southwest (Ibadan in Oyo State- 3,565,108), and Southeast Region (Owerri in Imo State- 215,038 and Abakaliki in Ebonyi State - 134,102) [13]. Furthermore, a large number of confirmed SARS-CoV-2 infection have been reported in each of these States during the first wave of COVID-19 [10]. Due to the high level of commercial activities in these areas, large open markets where a lot of people gather in the process of buying and selling were randomly selected in each of the States.

**Sample size:** based on assumed 50% prevalence of SARS-CoV-2 virus Infection, 5% significance level, we estimated minimum of 384 individuals in each State. Due to nature of the study area (open marketplaces), we couldn't develop a sampling frame. We therefore resulted into congruent enrolment of all consenting individuals until the minimum number is enrolled.

**Sample collection and processing:** discussions were held with market leaders who served as gate keepers to intimate them on the objectives of the study and then seek their permission to carry out the study in the market. A shade/spot was assigned to the study team by the market leaders for sample collection. Group pre-test counselling was provided and all those who gave consent were enrolled for the study and assigned unique identification numbers. The study was carried out between 20^th^ December, 2020 and 20^th^ March, 2021, a period that coincide with the second wave of the COVID-19 pandemic. Nasopharyngeal and oropharyngeal swabs sample were collected from each study participant according to the approved Nigeria Centre for Disease Control (NCDC) sample collection protocol. Samples were collected into appropriately labelled sterile tubes containing 1-2 ml of viral transport media (VTM). These were then wrapped in an adsorbent material that can absorb the content in the event of breakage or spillage and then placed in a leak-proof secondary container which is in turn placed in a zip-lock bag before being held in another airtight, sturdy container. This was then transported in a cold box containing frozen gel packs or ice packs to the laboratory were samples were processed immediately or stored at -80 °C until tested.

**Inclusion criteria:** individuals 15 years and above who gave informed consent and children (<15 years) whose parent/guardian requested that they participate in the study and gave ascent to be part of the study were enrolled.

**Exclusion criteria:** pregnant women and children not accompanied by their parent/guardian were excluded in the study.

**Laboratory analysis:** samples were analysed in laboratories accredited by the NCDC for SARS-CoV-2 PCR testing in the respective States where samples were collected except for Imo State where the samples were shipped to the Department of Virology, College of Medicine, University of Ibadan for testing. This is because unlike the others States where RT-PCR was used for diagnosis, GenXpert was the method of diagnosis in Imo State. These laboratories received supplies of appropriately validated nucleic acid extraction kits and real time RT-PCR kits from the NCDC at various time during the duration of this study. Upon sample arrival in the laboratory, RNA was extracted using RNA purification kit following manufacturers instruction. The extraction kits used were the DaAn Gene RNA/DNA Purification Kit (DaAn Gene Co, Ltd., of Sun Yat-sen University, Guangdong, P.R.C), Liferiver RNA isolation kit (Liferiver Biotech, Shanghai, China) and ELiGene (Elisabeth Pharmacon, Ceska Repiblika). Following RNA isolation reverse transcription polymerase chain reaction (RT-PCR) was carried out using the TaqPath 1-step RT-qPCR Mastermix (Thermo Fisher Scientific, Waltham, MA, USA), GeneFinder^™^ COVID-19 PLUS RealAmp Kit (OSANG. Healthcare Co., Seongnam, Korea) and ELiGene qPRC kit (Elisabeth Pharmacon, Ceska Repiblika) according to manufacturer's instruction.

**Data processing and analysis:** data analysis was done using Microsoft Excel and STATA. Descriptive statistics including mean and percentages were used. Chi-square tests of association were conducted between the participants' characteristics and SARS-CoV-2 infection results. The test of significance was set at P<0.05.

**Ethical approval statement:** ethical approval was obtained from the respective State ministries of health where this research was conducted.

**Funding source:** this work was supported by the Tertiary Education Trust Fund (TETFund) special COVID-19 Research Grant.

## Results

**Socio-demographic characteristics of participants:** a total of 2,158 participants were enrolled over the study period with males having the higher representation (55.5%). The mean age of the study participants was 38.4 (0.31) years. Table 1 shows the demographic characteristics of the study participants. Majority of the participants had at least a secondary school education (71%), were Muslims (70%), of the Igbo ethnic group (45%) and married (65.5%). Trading was the predominant occupation (50%) among those sampled in this study.

**Table 1:** demographic characteristics of study participants

State		Ebonyi	Imo	Kaduna	Kano	Oyo	Total
**Gender**	**(F:M)%**	44.7:54.3	63.1:36.9	30.4:69.6	12.5:87.5	57.9:42.1	44.5:55.5
**Age(years) Mean**		36.6	41.3	37.1	29.0	43.7	38.4
**Level of Education**	**(N:P:S:HS)**	0.4:21:56:23	2:19:47:32	2:38:26:34	1:31:43:25	9:31:46:15	3:27:44:26
**Religion**	**(C:I)**	98.91:1.09	99.56:0.44	7.49:92.51	0.00:100	29.9:70.1	47.17:52.83
**Ethnicity**	**(Fu:Ib:Yu:O)**	1:96:1:1	0.2:98.8:0:1	91:1:4:4	97.6:0:0.4:2	13:2:84:1	34:45:19:2
**Marital status**	**(CM:D:Sn)**	69.5:0.4:30.1	62.3:8.8:28.9	67.2:2.0:30.8	39.1:1.6:59.3	76.0:10.3:13.7	65.5:4.7:29.8
**Occupation**	**(Ar:Cs:St:Tr:Ue:O)**	6:16:13:52:6:6	5:13:14:47:4:14	1:33:16:12:4:21	3:2:22:69:1:2	13:1:2:80:0:4	6:14:12:50:3:10
**What are you doing?**	**(S:B)**	35.0:65.0	43.5:56.5	22.5:77.5	67.0:33.1	84.2:15.8	50.4:49.6

M=male F=female SE= standard error N=none P=primary S=secondary HS=higher than secondary fu-fulani Ib=igbo Yo=yoruba O=others Cm=currently married D=divorced Sn=single/never married Ar=artisan Cs-civil servant St=student Tr=trader Ue=unemployed, S=selling, B=buying

**Rate of infection among participants:** out of the 2,158 samples tested, 163 were positive, giving an overall prevalence of 7.6%. Table 2 shows the distribution of active SARS-CoV-2 infection by location of participants. The rate of infection was highest in the North-western zone (11%) and lowest in the South-eastern zone (4.9%). This difference in rate of infection across the geo-political zones was statistically significant (P=0.000, X^2^ =36.7017). Although 2,158 individuals participated in the study, only 1,830 had complete information/parameters hence were used for subsequent analysis.

**Table 2:** distribution of SARS-CoV-2 infection by location of participants

Zone	State	No Tested	No (%) Positive	No (%) Negative
Southeast	Ebonyi	498	30 (6.0)	468 (94.0)
	Imo	390	13 (3.7)	377 (96.7)
Subtotal		888	43 (4.9)	845 (95.2)
Northwest	Kaduna	492	50 (10.2)	442 (89.8)
	Kano	298	42 (14.1)	256 (55.9)
Subtotal		790	92 (11.6)	698 (88.4)
Southwest	Oyo	480	28 (5.8)	452 (94.2)
Subtotal		480	28 (5.8)	452 (94.2)
Total		2158	163 (7.6)	1995 (92.4)

Chi-square test of association: P=0.0001, X^2^ =36.7017

**Figure 2:**
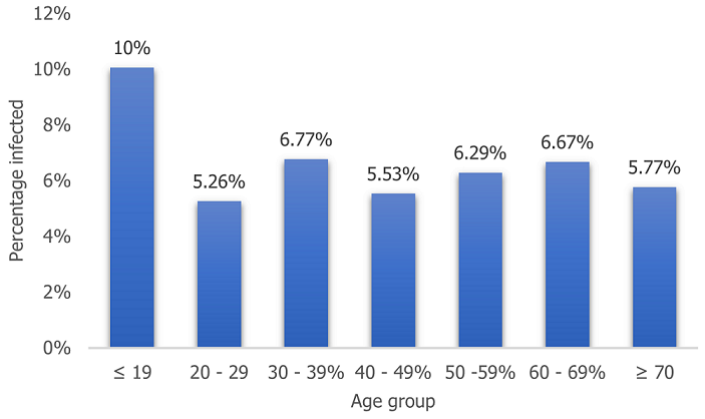
distribution of SARS-CoV-2 infection by age group of participants

**Rate of infection by gender and age of participants:** although there was no significant difference in the number of male and female participants enrolled for the study, the rate of SARS-CoV-2 infection was significantly higher (P=0.031, X^2^=4.6394) among males (7.6%). Similarly, the highest prevalence of infection (10%), was found among the ≤19 age group (Figure 2) however, the difference in prevalence among age group was not statistically significant (P=0.550, X^2^=4.9485).

**Rate of infection associated with other socio-demographic factors:** Table 3 shows the distribution of SARS-CoV-2 by education, religion, ethnicity and marital status. The rate of infection was highest among those with higher than secondary school education, Islam, other ethnic groups (followed by Hausa/Fulani), and the single/never married group. However, the difference in prevalence was only significant in the grouping by ethnicity (P=0.003).

**Table 3:** distribution of SARS-CoV-2 infection by education, religion, ethnicity and marital status of study participants

Variables	Category	No Tested	No (%) Positive	No (%) Negative	P value*
**Education**					
	None	59	4(6.8)	55(93.2)	
	Primary	490	30(6.1)	460(93.9)	0.060
	Secondary	809	41(5.1)	768(94.9)	
	Higher than secondary	472	42(8.9)	430(91.1)	
**Religion**					
	Christianity	1024	56(5.5)	968(94.5)	0.081
	Islam	803	60(7.5)	743(92.5)	
**Ethnicity**					
	Fulani/Hausa	524	47(9.0)	477(91.0)	
	Igbo	848	41(4.8)	807(95.2)	0.003
	Yoruba	416	23(5.5)	393(94.5)	
	Others	42	6(15.4)	36(84.6)	
**Marital status**					
	Currently married	1262	80(6.3)	1182(93.7)	
	Divorced	98	2(2.0)	96(98.0)	0.137
	Single/never married	470	35(7.5)	435(92.5)	

* Chi-square test of association

Occupation of the participants included trading, artisans, civil servants, students, house wives, the unemployed and others (Table 4). Although the rate was highest among students (8.9%) and lowest among traders (4.6%), the difference by occupation was not significant (P=0.122). The rate of infection was however significantly higher (P=0.001) among the buyers (7.4%) than sellers (Table 4).

**Table 4:** distribution SARS-CoV-2 infection by occupation of participants and their roles at the market

	No Tested	No (%) Positive	No (%) Negative	P-value*
**Occupation**				
Artisan	119	9(7.6)	110(94.4)	0.122
Civil servant	302	24(8.0)	278(92.0)	
Housewife	87	7(8.1)	80(92.0)	
Student	202	18(8.9)	184(91.0)	
Trader	869	40(4.6)	829(95.4)	
Unemployed	59	3(5.1)	56(94.9)	
Others	192	16(8.3)	176(91.7)	
**Roles at the market**				
Selling	853	45(5.28)	808(94.7)	0.001
Buying	977	75(7.37)	905(92.6)	
Total	1830	117(**6.39**)	1713(93.6)	

* Chi-square test of association

## Discussion

Findings from this study indicates that 7.6% of Nigerians across three Geo-Political zones had ongoing SARS-CoV-2 infection during this study that coincided with the second wave of COVID-19 in the country. Interestingly, these individuals were asymptomatic as they did not report presence of any COVID-19 related health issues during the time of sample collection. This is disturbing considering the fact that the world at large is still trying to control the pandemic. Even more disturbing is the fact that Nigeria, with a national prevalence of about 10% (range 2.5% - 16%) at this time, as well as, many other African countries have developing health infrastructures that need to be protected from being overwhelmed [14]. The prevalence obtained in this study was higher than 5.6% global prevalence of SARS-CoV-2 virus Infection as at March, 2022 [13]. This shows that the infection rate is relatively high and drastic measures are needed to curb the rapid spread of the virus. On the other hand, the rate in this study is lower than rates obtained in a retrospective cohort study in Lagos (14.6%), the most populous city in Nigeria by Salako and colleagues [15] and 15.2% obtained among healthcare workers in Rivers State, Nigeria [16]. The difference in the rate of the infection in these studies and ours may be due to difference in the study population. While the Lagos and Rivers State studies were among high-risk individuals (COVID-19 suspects and health care workers respectively), our study was among apparently healthy asymptomatic individuals.

As at 16^th^ May, 2022, data from the Nigeria Centre for Disease Control indicates that over 255,000 cases have been confirmed and over 5.1 million samples have been tested [10]. This further underscore the need for studies such as this, which spans across various geopolitical zones of the country and targets an active subset of the population in urban centers. The results of this study however raised a fundamental question that requires further investigation. That is, why the level of SARS-CoV-2 Infection in the community is not commensurate with the number of COVID-19 cases and deaths in the country.

The rate of infection found in this study shows that the spread was significantly higher in the northern part of the country. However, another study showed that youths who had tertiary education in the Northern western part of the country displayed higher levels of awareness about the mode of spread of the virus when compared to their counterparts from the southern and western parts of the country [17]. The higher rate of infection recorded in this study may be due to the fact that majority of the participants were not adhering to recommended safety protocols. There is therefore a need for more enforcement of these protocols, most especially in public places as much as possible.

The rate of infection among males were significantly higher than that found among females. This finding corroborates those of other researchers, who earlier reported higher morbidity and mortality among males than females [18,19]. This has been linked to the habit of smoking and alcohol consumption, which is more predominant among the male gender. It is common for alcoholics in pursuit of their indulgences not to stay at home. They often sit together with other people without social distancing and remove their mask to drink and smoke [18,20]. Other factors that have been associated to this trend include, higher expression of angiotensin-converting enzyme-2 receptors for coronavirus [21], sex-based immunological differences driven by sex hormone and X chromosome [22,23]. Furthermore, females have been reported to exhibit more responsible attitude toward the COVID-19 pandemic than men [24]. Participants who were less than 20 years old had the highest (10%) positivity rate for the infection. A similarly trend of infection among this age group has been reported in Lagos [15]. Although, most children with the virus infection are usually asymptomatic or have mild symptoms [25] due to factors such as lower expression and functioning of ACE2 receptors and their recurrent exposure to viral infections, thereby modulating their response to SARS-CoV-2 virus [26,27], their role in serving as a source of exposure and infection to older and immunocompromised individuals should not be overlooked.

Our results also showed that SARS-CoV-2 virus infection was higher among those that had post-secondary education. This is surprising as an educated group is expected to have more information on preventive measures against the virus [28,29]. This observation may point to the fact that inadequate adherence, rather than an absence of information may be responsible for the spread of the disease. Therefore, it is necessary to establish some system of enforcement of safety practices as much as possible in the community. Also, individuals with higher level of education are more likely to be working in an enclosed space (office) with higher risk if transmitting the virus, if introduced, as well as, participants in parties and other large gatherings with in an enclosed space. A higher rate of infection was also found among Muslim participants in our study, there is a need to involve regions clerics on need to encourage their members to protect themselves from the virus. They should also discourage religious events that results in the congregation of a large number of people in the same place. Study participants who were single also had the highest rate of infection. Although the rate of infection was not statistically different by marital status, the rate was slightly higher among the single/never married. These single/never married group also includes students who also had a high rate of infection in this study. Some studies have shown that young people are less likely to adhere to the non-pharmaceutical preventive measures. In addition, they are more active and restless and are more likely to visit places where they will become more exposed and infected. Previous studies have shown that Vitamin D is vital in regulating the immune system and that the exposure of the skin to sunlight UV radiation produces Vitamin D [30], it is possible that the lower level of infection observed among sellers is due to the fact that sellers in the open market have a high level of exposure to sunshine while carrying out their business.

**Limitations:** due to limited funding, only 3 of the six geo-political zones in Nigeria were included in the study. Focus on marketplaces, the purposive selection of study areas and the congruent recruitment of participants did not give equal probability of participation to all Nigeria residents. Therefore, the data used for this study is not nationally representative. However, the approach has a strength in that it focused on large gathering of people where SARS-CoV-2 virus is easily shared.

## Conclusion

This study has shown the prevalence of SARS-CoV-2 virus across different geographical zones in Nigeria. It has also made it clear that a higher rate of infection was going on at the community level when compared with information from the national database due to insufficient testing. Of interest is the fact that these individuals were asymptomatic with no COVID-19 related illness. It thus brought to fore the need to further investigate the reason for lower COVID-19 case and COVID related death even when the rate of SARS-CoV-2 infection among apparently healthy individuals in the community is high.

### What is known about this topic

- *Globally, The SARS-CoV-2 pandemic caused significant morbidity and mortality in human population;*
- *Africans experienced a lower number of mortalities from the SARS-CoV-2 pandemic*.

### What this study adds

- *During the pandemic, ongoing SARS-CoV-2 infection among Nigerians was higher than what was reported in the national database;*
- *Higher infection rate was found in the Northwestern States of Nigeria*.
